# Supplementary material for: Phosphorylation but Not Oligomerization Drives the Accumulation of Tau with Nucleoporin Nup98
Source: Int J Mol Sci. 2022 Mar 23;23(7):3495. doi: 10.3390/ijms23073495 (PMC8998617; doi:10.3390/ijms23073495)
Supplement: Supplementary file 1 [file ijms-23-03495-s001.zip › ijms-1623190-supplementary.pdf]

International Journal of *Molecular Sciences*

MDPI

Article

Phosphorylation but Not Oligomerization Drives the Accumulation of Tau with Nucleoporin Nup98

Lisa Diez [1], Larisa E. Kapinos [2], Janine Hochmair [1], Sabrina Huebschmann [1], Alvaro Dominguez-Baquero [1], Amelie Vogt [1], Marija Rankovic [3], Markus Zweckstetter [3,4], Roderick Y. H. Lim [2] and Susanne Wegmann [1,*]

Supplemental information

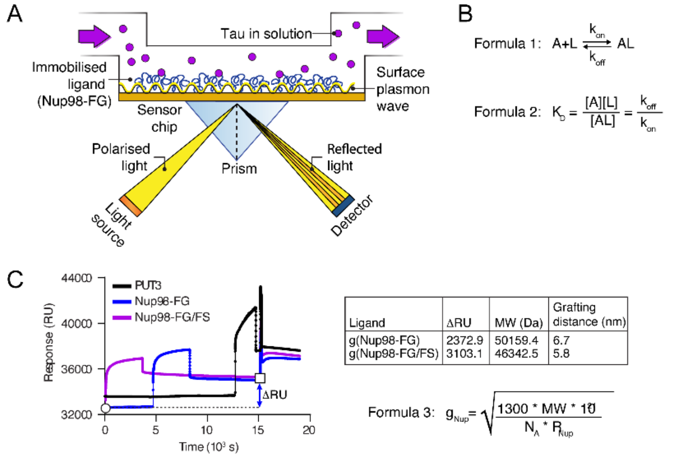

D

Nup98-FG (human; aa 1-498)

```
MFNKSFGTPF GGGTGGFGTT STFGQNTGFG TTSGGAFGTS AFGSSNNTGG
LFGNSQTKPG GLFGTSSFSQ PATSTSTGFG FGTSTGTANT LFGTASTGTS
LFSSQNNAFA QNKPTGFGNF GTSTSSGGLF GTTNTTSNPF GSTSGSLFGP
SSFTAAPTGT TIKFNPPTGT DTMVKAGVST NISTKHQCIT AMKEYESKSL
EELRLEDYQA NRKGPQNQVG AGTTTGLFGS SPATSSATGL FSSSTTNSGF
AYGQNKTAFG TSTTGFGTNP GGLFGQQNQQ TTSLFSKPFG QATTTQNTGF
SFGNTSTIGQ PSTNTMGLFG VTQASQPGGL FGTATNTSTG TAFGTGTGLF
GQTNTGFGAV GSTLFGNNKL TTFGSSTTSA PSFGTTSGGL FGNKPTLTLG
TNTNTSNFGF GTNTSGNSIF GSKPAPGTLG TGLGAGFGTA LGAGQASLFG
NNQPKIGGPL GTGAFGAPGF NTTTATLGFG APQAPVALTD PNASAAQQAV
LQQHINSLTY SPFGDS
```

Nup98-FG F-S mutant (human; aa 1-498)

```
MSNKSSGTPS GGGTGGSGTT STSGQNTGSG TTSGGASGTS ASGSSNNTGG
LSGNSQTKPG GLSGTSSSSQ PATSTSTGSG SGTSTGTANT LSGTASTGTS
LSSSQNNASA QNKPTGSGNS GTSTSSGGLS GTTNTTSNPS GSTSGSLSGP
SSSTAAPTGT TIKSNPPTGT DTMVKAGVST NISTKHQCIT AMKEYESKSL
EELRLEDYQA NRKGPQNQVG AGTTTGLSGS SPATSSATGL SSSSTTNSGS
AYGQNKTASG TSTTGSGTNP GGLSGQQNQQ TTSLSSKPSG QATTTQNTGS
SSGNTSTIGQ PSTNTMGLSG VTQASQPGGL SGTATNTSTG TASGTGTGLS
GQTNTGSGAV GSTLSGNNKL TTSGSSTTSA PSSGTTSGGL SGNKPTLTLG
TNTNTSNSGS GTNTSGNSIS GSKPAPGTLG TGLGAGSGTA LGAGQASLSG
NNQPKIGGPL GTGASGAPGS NTTTATLGSG APQAPVALTD PNASAAQQAV
LQQHINSLTY SPSGDS
```

Figure S1. SPR principle, binding kinetics, and Nup98-FG layer immobilization. (**A**) Scheme of SPR experimental setup. The SPR sensor detects the binding of an analyte (e.g. Tau) in solution to a ligand (e.g. Nup98-FG) immobilized on the gold surface of the sensor chip. This binding alters the refractive index of the medium near the surface, which is monitored by a detector. (**B**) Formula 1

and Formula 2 for binding kinetics. (**C**) Immobilization of gold sensor chip for experiment shown in Figure 1F. In the shown example, the grafting distance, which gives information on how spares/dense the Nup98-FG layer is packed, is similar for the analyzed Nup98 variants (Nup98-FG and Nup98-FG/FS mutant). The grafting distance g(Nup98-FG) is calculated using the indicated formula with NA being the Avogadro constant, MW the molecular weight of Nup98-FG, and ΔRU the difference in SPR response before (white circle, t = 0 s) and after (white square, t = $15x10^3$ s) Nup98-FG immobilization. ΔRU of 1300 equals 1 ng Nup98-FG/mm^2 sensor chip surface. (**D**) Peptide sequence of Nup98-FG and Nup98-FG F/S mutant.

A

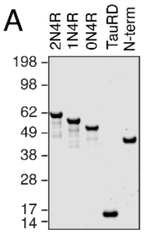

B

	Nup98-FG	2N4R	1N4R	0N4R	TauRD	N-term
Molecular weight (g/mol)	49169.23	45850	42966.9	40007	13814	26457
Isoelectric point	10	8.6	9.4	9.9	10.2	5.2
Net charge at pH 7.0	7.1	3.1	7	13.9	10.4	-5.6
Average hydrophilicity	-0.3	0.4	0.4	0.4	0.3	0.5
Ratio hydrophilic aa/ tot aa	0.29	0.43	0.44	0.43	0.45	0.41

C

Nup98-FG	K_D (M)	k_{on} (1/Ms)	k_{on} Error	k_{off} (1/s)	k_{off} Error	R_{max}
2N4R	8.546E-7	1.081E+5	4.524E+3	9.234E-2	2.83E-3	2.4570
TauRD	5.396E-7	1.697E+5	4.192E+3	9.156E-2	1.485E-3	0.4734
N-term	1.251E-6	1.749E+4	9.008E+2	2.188E-2	8.472E-4	0.0851

D

Nup98-FG	K_D (M)	k_{on} (1/Ms)	k_{on} Error	k_{off} (1/s)	k_{off} Error	R_{max}
2N4R	2.756E-5	1.736E+3	5.044E+2	4.784E-2	1.379E-3	0.5451
1N4R	1.755E-5	7.749E+4	8.524E+4	1.360E+0	2.748E-1	0.1538
0N4R	1.473E-5	1.542E+4	4.451E+3	2.272E-1	1.135E-2	0.398
TauRD	2.723E-5	2.115E+3	6.857E+2	5.759E-2	1.908E-3	0.4073
N-term	4.126E-2	3.632	6.987E+3	1.499E-1	2.252E-2	51.85

Figure S2. Biochemical and binding kinetic data for Tau variants. (**A**) Coomassie gel of recombinant expressed Tau variants. (**B**) Protein biochemical properties of Tau variants. (**C**) Association (k_{on}) and dissociation constants (k_{off}) for Tau 2N4R, TauRD, and N-term binding to Nup98-FG. Values derived from fits to the data shown in Figure 2D. (**D**) Association (k_{on}) and dissociation constants (k_{off}) for Tau isoform and domain binding to Nup98-FG. Values derived from fits to the data shown in Figure 2F.

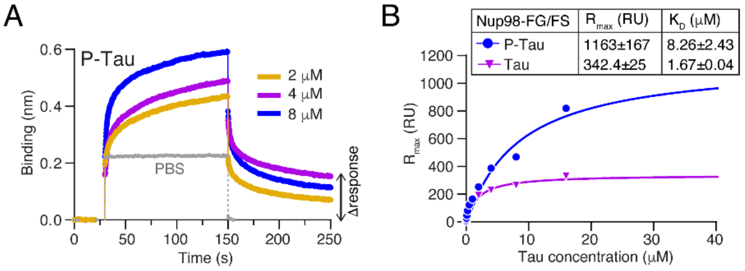

Figure S3. Binding kinetics of P-Tau to Nup98-FG/FS. (**A**) BLI measurement of P-Tau (2, 4 and 8 μM) binding to Nup98-FG compared to PBS (grey). The accumulation of P-Tau on the Nup98-FG layer is apparent from the incomplete dissociation (Δresponse). (**B**) Equilibrium fit (Langmuir binding isotherm) to RU_{max} plotted versus Tau concentration for the binding of Tau and P-Tau to mutant Nup98-FG/FS. In the Table, fit results are shown as value±SD.

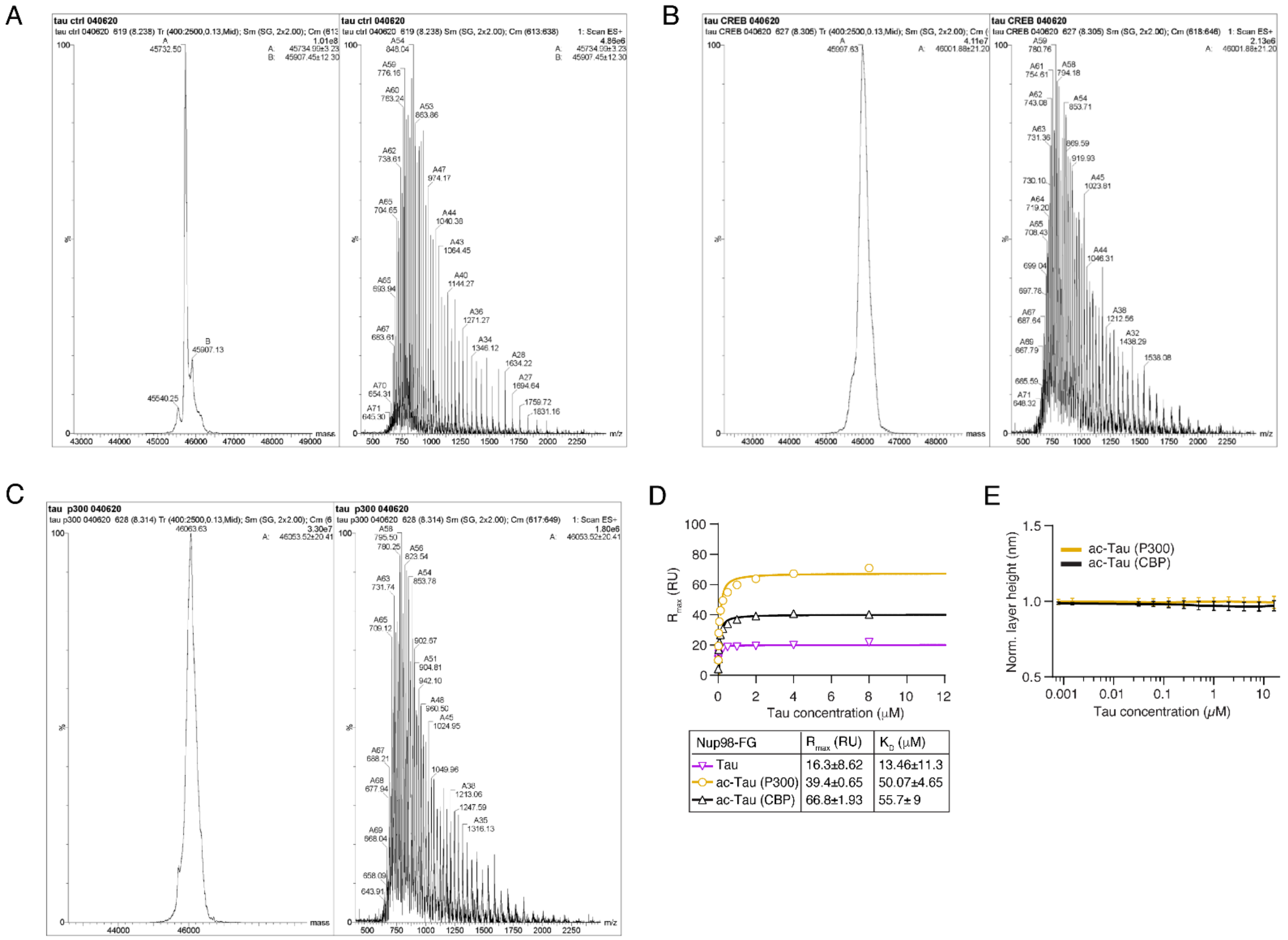

Nup98-FG	R_{max} (RU)	K_D (μM)
Tau	16.3±8.62	13.46±11.3
ac-Tau (P300)	39.4±0.65	50.07±4.65
ac-Tau (CBP)	66.8±1.93	55.7± 9

Figure S4. Mass spectrometry analysis of Tau acetylation and binding kinetics of ac-Tau to Nup98-FG. (**A-C**) Mass spectra of unmodified (**A**), CBP acetylated (**B**) and P300 acetylated Tau (**C**). Identified masses (in Da) are indicated showing Tau acetylatilation at 6-8 sites. (**D**) Equilibrium fit (Langmuir binding isotherm) to RU_{max} (from SPR measurements) plotted *versus* Tau concentration for the binding reactions of acetylated Tau compared to unmodified Tau. Tau was acetylated *in vitro* using P300 or CBP. In the Table, fit results are shown as value±SD. (**E**) Layer height analysis of *in vitro* acetylated Tau (using P300 or CBP) shows no accumulation of Tau on the Nup98-FG layer.

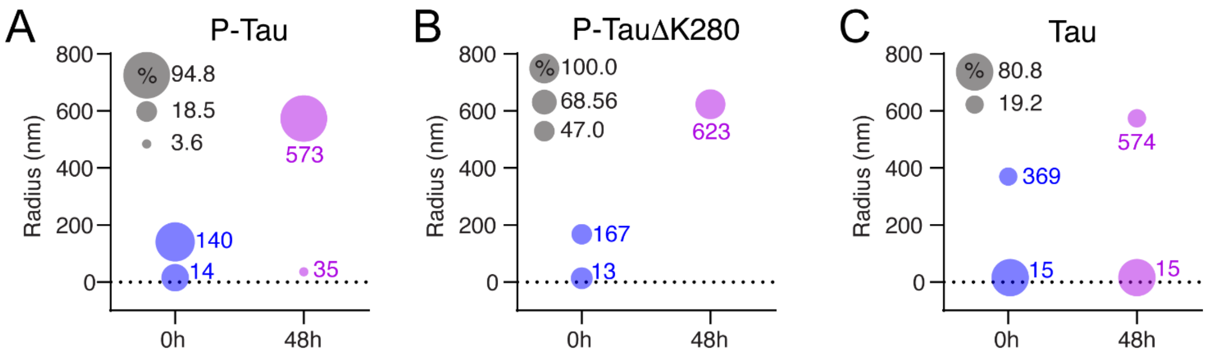

Figure S5. Radii of Tau measured by DLS before (0h) and after (48h) oligomerization. (**A-C**) Bubble plots of particle sizes for P-Tau (**A**), P-TauΔK280 (**B**) and Tau (**C**) before (0 h, blue) and after (48 h, purple) incubation at room temperature to enable oligomerization. Bubble sizes correspond to the %-age of different radius populations present (same data as shown in Figure 4A), and numbers in bubbles indicate the mean radius in nm.. The majority of P-Tau and P-TauΔK280 particles have a larger radius after 48h, indicating their oligomerization. Most Tau particles remain small, indicating a monomeric state.
